# Effect of Intensity and Duration of Exercise on Gut Microbiota in Humans: A Systematic Review

**DOI:** 10.3390/ijerph19159518

**Published:** 2022-08-03

**Authors:** Romina Bonomini-Gnutzmann, Julio Plaza-Díaz, Carlos Jorquera-Aguilera, Andrés Rodríguez-Rodríguez, Fernando Rodríguez-Rodríguez

**Affiliations:** 1 Escuela de Nutrición y Dietética, Facultad de Ciencias, Universidad Mayor, Santiago 8580745, Chile; rominabonomini@gmail.com; 2 Children’s Hospital of Eastern Ontario Research Institute, Ottawa, ON K1H 8L1, Canada; 3 Department of Biochemistry and Molecular Biology II, School of Pharmacy, University of Granada, 18071 Granada, Spain; 4 Instituto de Investigación Biosanitaria IBS.GRANADA, Complejo Hospitalario Universitario de Granada, 18014 Granada, Spain; 5 Gastric Cancer Research Group—Laboratory of Oncology, UC Center for Investigational Oncology (CITO), Pontificia Universidad Católica de Chile, Santiago 8331150, Chile; airodriguez1@uc.cl; 6 IRyS Group, Physical Education School, Pontificia Universidad Católica de Valparaíso, Valparaíso 2374631, Chile; fernando.rodriguez@pucv.cl

**Keywords:** aerobic exercise, adults, elite athletes, large intestine, gut microbiota

## Abstract

(1) Background: The gut microbiota might play a part in affecting athletic performance and is of considerable importance to athletes. The aim of this study was to search the recent knowledge of the protagonist played by high-intensity and high-duration aerobic exercise on gut microbiota composition in athletes and how these effects could provide disadvantages in sports performance. (2) Methods: This systematic review follows the PRISMA guidelines. An exhaustive bibliographic search in Web of Science, PubMed, and Scopus was conducted considering the articles published in the last 5 years. The selected articles were categorized according to the type of study. The risk of bias was assessed using the Joanna Briggs Institute’s Critical Appraisal Tool for Systematic Reviews. (3) Results: Thirteen studies had negative effects of aerobic exercise on intestinal microbiota such as an upsurge in I-FABP, intestinal distress, and changes in the gut microbiota, such as an increase in *Prevotella*, intestinal permeability and zonulin. In contrast, seven studies observed positive effects of endurance exercise, including an increase in the level of bacteria such as increased microbial diversity and increased intestinal metabolites. (4) Conclusions: A large part of the studies found reported adverse effects on the intestinal microbiota when performing endurance exercises. In studies carried out on athletes, more negative effects on the microbiota were found than in those carried out on non-athletic subjects.

## 1. Introduction

The intestinal or gut microbiota is “the set of microbes that colonize our digestive tract that interact with each other and with the host” [1,2,3]. Currently, more than one thousand different microbial species have been found that can reside in the human gastrointestinal tract [4]. Approximately one hundred sixty species are found in the large intestine [3], developing a biomass of more than 1.5 kg [5]. The microbiota contain bacteria, as well as fungi, viruses, and protists [1]. The most abundant and diverse families of bacteria in the adult gastrointestinal tract are *Actinobacteria*, *Bacteroidetes*, *Firmicutes*, and *Proteobacteria*, and less diverse are *Verrucomicrobia*, *Lentisphaerae*, *Sinergistetes*, *Planctomycetes*, *Tenericutes*, and *Deinococcus-Thermus* [2,3].

The composition of the gut microbiota is formed throughout early childhood, induced by genetic and environmental factors [1,2,3]. The maturation of the intestinal microbiota in the adult type is gone at the age of three years [1,6]. Factors such as age, lifestyle, diet, and genetics can alter the gut microbiota, creating a dynamic ecosystem [7]. Other related factors could include mode of delivery, geography, breastfeeding, weaning, and exposure to environmental bacteria [1]. Some researchers proposed that the intestinal microbiota can act as an endocrine organ [8] and can have an enormous impact on human health, including the immune function, physiology, metabolism, and nutrition of the host [9]. In the same line, the gut microbiota performs a series of protective, structural, and metabolic functions essential to the health of the host, including food handling, the ingestion of complex polysaccharides not digestible by the host, the movement of pathogens, and the synthesis of vitamins among others [10]. Observational studies have found that intestinal microbiota may contribute either to the pathogenesis of various common metabolic disorders including type 2 diabetes, obesity, cardio-metabolic diseases, malnutrition, and non-alcoholic liver disease, as well as to the metabolic health of the human host [11]. Healthy gut microbiota shows an essential role in the configuration of the local and systemic immune function of intestinal bacteria throughout life, favoring the maintenance of tolerance toward antigens of the commensals and activation against antigens of commensal pathogens [12]. The intestinal microbiota plays an important role in the regulation of host energy metabolism, hydration status, systemic inflammatory responses, and oxidative stress [13].

Physical exercise is described as the implementation of some activity in order to improve or preserve overall health and physical fitness [14,15]. Currently, physical exercise is recognized as a formidable preventive and treatment mediation that is recognized to be efficient in causing benefits for immune and metabolic health [15,16]. Endurance exercise is described as cardiovascular activity, for example: cycling, running, swimming, skiing, and rowing that is performed for a long time, four to six hours per day, six days per week [17]. This intense exercise includes processes that involve physiological, affective, cognitive–behavioral, and biochemical responses in an effort to recover homeostasis [9].

Some professional athletes experience immunosuppression or gastrointestinal symptoms, such as abdominal pain, diarrhea, or leaky gut syndrome [8]. Alterations in the intestinal microbiota produced by strenuous exercise can also produce exercise-induced gastrointestinal disorders [18]. Some symptoms that are stated during the performance of endurance exercise include bloating, nausea, cramps, and diarrhea [19]. It has been studied that exercising to exhaustion can disrupt the balance among the gut microbiota and the immune system [12]. Exercise-induced gastrointestinal damage or inflammation could adversely affect athletic routine and, in some cases, have competition dropout [20]. Another study mentions that intense exercise creates increased gastrointestinal damage, mild endotoxemia, and intestinal permeability [21]. The main finding in post-exercise gastrointestinal problems is possible ischemia–reperfusion injury developing from a momentary interruption of splanchnic blood flow. When the intense physical exercise finishes, we observed triggering reactive oxygen species (ROS) production, damage to the gastrointestinal mucosa, and inflammation [22].

Likewise, it is little known thus far how high-intensity exercise influences the intestinal microbiota [21]. The importance of knowing the mechanisms in which the intestinal microbiota might have an important role in affecting athletic routine is of significant attention to athletes working to expand their competitive performance and diminish recuperation time during training [12]. Such information could have an advantage in the comprehension of gut microbiota influences on athlete health [12].

Therefore, the main aim of this systematic review is to elucidate the knowledge of the function played by high-intensity and high-duration aerobic exercise on gut microbiota composition in athletes and how these effects could provide disadvantages in their sports performance.

## 2. Materials and Methods

### 2.1. Search Strategy

This systematic review follows the PRISMA guidelines [23]. An exhaustive bibliographic search of three databases (Web of Science, PubMed, and Scopus) was conducted considering the articles published in the last 7 years (from 1 January 2015 to 31 August 2021). Table 1 shows the search strategy in the Web of Science, PubMed, and Scopus databases. This systematic review was listed on the PROSPERO (International prospective register of systematic reviews) website on 5 May 2022, with the following record CRD42022323300. Available from: https://www.crd.york.ac.uk/prospero/display_record.php?ID=CRD42022323300 (accessed on 14 June 2022).

### 2.2. Selection and Exclusion Criteria

The selection criteria were: (i) articles written in English, (ii) databases aforementioned, (iii) human studies, (iv) original articles: clinical trials, randomized controlled trials (RCTs) quasi-experimental, long-term, prospective, and cross-sectional studies, (v) articles from January 2015 to August 2021. The exclusion criteria were: (i) studies that include people with pathologies, (ii) studies that include animals, (iii) studies that comprise children under 18 years of age and older adults (+65 years), (iv) studies that intervened with supplements or some diet, (v) case studies, case reports, letters to the editor, systematic review and meta-analyses and narrative review. No restrictions were placed on the body composition of the trained subject.

After removing repeated documents, suitability was measured by evaluation of the manuscript title and abstract and later evaluation of the full text.

### 2.3. Data Extraction and Reliability

The search was carried out by five independent reviewers (R.B.-G., F.R.-R., J.P.-D., C.J.-A. and A.R.-R.). They read the titles and abstracts of all retrieved articles. A meeting was held to resolve disagreements about eligibility. The following information was collected from each included study: the first author, year of publication, type of study, objective, the number of subjects, body mass index (BMI), maximum oxygen consumption (VO_2max_), gender, and age when it was available, type of exercise, how the exercise was carried out, the molecular analysis used for the detection of the gut microbiota, the main results obtained, and conclusions.

The selected articles were categorized according to the type of study (low-, medium-, or high-intensity or long-term exercise interventions). The results of the studies that met the selection criteria for their recovery were examined.

### 2.4. Assessment of the Quality and Level of Evidence

The risk of bias was assessed using the Joanna Briggs Institute’s Critical Appraisal Tool for Systematic Reviews. In summary, this tool includes four specific checklists depending on the study design (i.e., cross-sectional, quasi-experimental, cohort, RCTs studies). The answers for each of them had four possible categories: “yes” (criterion met) and “no” (criterion not met). Specific tools included: eight items for cross-sectional studies, nine items for quasi-experimental, and thirteen items for RCTs. According to the above, the studies were considered as “low quality” evidence when ≤49% of the items were classified as “yes” (criterion met). Following, the articles were considered as “medium quality” evidence between 50–74% of the items were scored as “yes” and “high quality” evidence when ≥75% of the items were classified as “yes”. The answers “not applicable” and “non-clear” were excluded by percentage [24,25,26]. The five reviewers assessed the studies’ quality separately. A consensus meeting was organized to resolve possible differences between the reviewers.

## 3. Results

**Figure 1:**
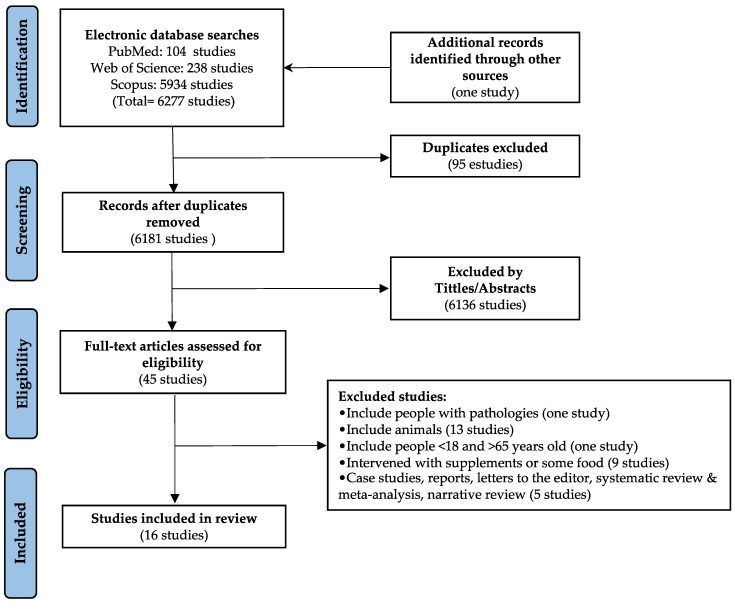
Flowchart of articles through the search process.

Figure 1 displays the chosen reporting elements for systematic reviews and the flow chart for the search strategy. A total of 6277 studies were located in the three databases assessed. Then, 95 studies were excluded for duplicates and 6136 studies were excluded after reading the title and abstract that were outside the topic of the review. A total of 45 studies were assessed for eligibility. After analyzing the exclusion criteria, sixteen studies were included. Seven studies had an observational design (i.e., five cross-sectional and two long-term designs), and nine studies had an experimental design (eight were quasi-experimental, and one was RCT).

Table 2 shows the summary of the studies included. This review is focused on data from 513 participants, and the sample size ranged from 4 to 86 subjects. Two of the sixteen studies involved only women [18,27], six involved only men [5,13,16,28,29,30], five involved both men and women [7,9,22,31,32] and three studies did not report the sex of the subjects [19,33,34]. The age of the subjects ranged from 18 and 49 years; two studies did not report the age of the subjects [28,33]. The samples were from 10 different countries: three studies were led in Poland, two in Spain, two in the United States, two in China, two in Ireland, one in the United Kingdom, one in Australia, one in Belgium, one in Germany and one from Japan.

Regarding the characteristics of the sample, eleven studies of the sixteen were conducted on endurance athletes (runners, cyclists, and triathletes). Among these nine studies considering medium- and long-distance runners [5,13,16,18,22,29,30,32,33], three studies incorporated physically active subjects [27,31,34], one study on triathletes [19], a study on cyclists [7], a study included martial arts professionals [9], and a study on rugby players [28]. Most of the studies incorporated in this systematic review (81%) used fecal samples to determine changes in the intestinal microbiota. Of these, mainly 75% determined the 16S ribosomal RNA genetic sequence that is commonly used for identification, classification, and quantitation of microbes within complex biological mixtures such as environmental samples and intestinal samples. A minority of four studies (25%) used plasma samples through the protein enzyme-linked immunosorbent assay test to determine markers such as intestinal fatty-acid binding protein (I-FABP) related to mucosal damage, zonulin associated with intestinal permeability, and cortisol, c-reactive protein, and TNF-α related to a proinflammatory status.

The results revealed that nine studies showed negative effects of aerobic exercise on the intestinal microbiota. Among these adverse effects, three studies found an increase in I-FABP [5,16,29], one study presented intestinal distress [5], three studies observed negative changes in the microbiome [30,31,32], two studies found an increase in *Prevotella* [7,33], three studies observed an increase in intestinal permeability [16,19,29], and two studies reported an increase in zonulin [19,29]. In contrast, seven studies observed positive effects of endurance exercise, including an increase in the level of bacteria such as *Roseburia hominis, Bifidobacterium* spp., *Akkermansia muciniphila*, *Faecalibacterium prausnitzii* [18,27], *Coriobacteriaceae* [22], increased microbial diversity [9,13,33] and increased intestinal metabolites [28].

Additionally, an analysis has been performed to determine the study qualities included in this review. For this, the Joanna Briggs Institute’s criterium checklist (Table 3) was used. Different criteria were used according to the characteristics of the studies. In this regard, one study fulfilled 100% of the criteria [27], and seven studies fulfilled ≥75% of the quality criteria [7,9,13,27,28,31,33], classifying themselves as high quality. The rest of the studies [5,16,18,19,22,29,30,32,34] were classified as medium quality because they obtained a value between >50% and <75% of the criteria (among 50–69.2%). No studies with low quality were found (<50% of the criteria).

## 4. Discussion

The main aim of this study was to elucidate the recent knowledge of the function played by high-intensity and high-duration aerobic exercise on gut microbiota composition in athletes and how these effects could provide disadvantages in sports performance.

Since the beginning of the metagenomic era, microbial communities have been associated with human health [35]. We have described that the microbiota, the full collection of microbes, are important in the development of several diseases [36]. However, the presence of these microbes is not the only factor that affects the host. The genetics of all the microbes (bacteria, fungi, protozoa, and viruses), defined as the microbiome, and the metabolic products that they produce are other sources of important changes [37]. Centenarians have shown specific intestinal microbiota that are improved in microbes that are efficient in generating exclusive secondary bile acids comprising different lithocholic acid isoforms: isoallolithocholic acid, iso-, 3-oxo, allo-, and 3-oxoallo- [38].

The metabolic catalog of the intestinal microbiome is immense, but the well-being associations of these bacterial pathways is weakly comprehended. In the case of physical exercise, *Veillonella atypica* increases run time in humans via its metabolic conversion of exercise-induced lactate into propionate, thus recognizing a natural, microbiome-encoded enzymatic process that enhances athletic performance [39].

Following these lines, the main topics in the present systematic review were: (i) the strategies of analysis, (ii) the effects of exercise duration on microbiota, and finally, (iii) the effects of exercise intensity on the microbiota.

### 4.1. Strategies of Analysis

These microbes could be analyzed by several molecular techniques, for example using probes that detected a long range of bacterial strains [40]. This approach is more general, in which microbes could be presented in the sample, and is less effective in an individual and precise identification. In addition, another alternative is using the conserved 16S ribosomal RNA gene [41], which allows us better sensibility and precision in identification. These methods are joined with plenty of bioinformatic tools and processes [42]. Finally, other biochemical techniques such as assessment of intestinal permeability and gastrointestinal discomfort were used.

Since the initiation of high-throughput sequencing, PCR-amplified 16S sequences have habitually been gathered based on similarity to produce operational taxonomic units (OTUs), amplicon sequence variants (ASVs), and these descriptive sequences compared with reference databases such as Silva [43] or Greengenes [44], and Ribosomal Database Project (RDP) [45] to extrapolate the taxonomy [46].

In the total of 16 studies, we found that the majority of the studies used the 16S rRNA technique and metagenomic approaches (12/16; 75.0%), which ensure strong identification of the microbes that are presented in the different samples. The remaining studies (4/16; 25.0%) have reported secondary values, such as intestinal permeability or the release of different proteins, which could show a general perspective about gut microbiota.

The conclusions that were based on 16S and metagenomic approaches were more consistent and allowed the investigators to attribute the effects or not to individual genera or singular strains. Another important issue in the strategies of analysis was the type of study. Here, we have eight quasi-experimental studies, five cross-sectional studies, two long-term studies, and one RCT.

### 4.2. Effects of Exercise Duration on Microbiota

Physical activity, exercise, or physical fitness are considered advantageous therapies to decrease inflammatory pathways [47]. Recent evidence proposes that exercise can positively modify the intestinal microbiota composition in healthy adults [47,48]. These changes can also be discovered in patients with inflammatory bowel disease. Exercise programs of at least 12 weeks produce modifications in the gut microbiota composition through immunometabolic pathways associated with anti-inflammatory effects [49].

A recent systematic review has found that *Prevotella* relative abundance looks to be associated with training duration [50]. In our systematic review, we found a similar result with a high *Prevotella* abundance associated with time-reported exercising during an average week. In addition, the authors have found that *Methanobrevibacter smithii* transcripts were more predominant in professional cyclists in comparison to amateur cyclists [7]. In the same line, a higher *Faecalibacterium* abundance was found in the intestinal microbiota of female elite endurance runners related to the accumulation of succinate [18]. Marathon runners have shown increased levels of *Prevotella* and bacterial diversity [33] and alterations in the intestinal microbiota related to *Coriobacteriaceae* [22]. Both *Faecalibacterium* and *Prevotella* are related to human health benefits [51,52] and to plant-rich diets characterized by high levels of complex carbohydrates and vegetable and fruit intake [51,52]. The family *Coriobacteriaceae* may partly mediate the positive effects of Roux-en-Y gastric bypass on type 2 diabetes [53].

According to the secondary variables related to the gut microbiota, the studies have shown that running induced an upsurge in serum I-FABP concentration and intestinal permeability, but there were no differences between asymptomatic and symptomatic runners [16]. Serum LPS activity did not change from baseline following the running test, but the symptomatic group exhibited higher LPS activity at baseline compared to the asymptomatic runners [16]. Twelve-hour runs would provoke metabolic stress in middle-aged amateur runners and elevated levels of biomarkers such as zonulin, hs-CRP, and I-FABP [29]. Endurance-sport athletes have excessive gastrointestinal disorders prevalence and bargaining performance, possibly affecting general health status. Ultramarathoners and triathlon athletes have shown an increase in several proinflammatory cytokines and proteins [54]. The analyzed studies have shown a pro-inflammatory status related to intestinal microbiota, similar to the recent systematic review that states that exercise duration could be related to a higher pro-inflammatory bacteria abundance [50,55,56].

Exercise is a powerful intervention to fight obesity that is also related to a proinflammatory status and poorer vascular function [57]. Studies relating to intestinal microbiota changes have stated that exercise could raise the microbial variance and enhance the *Firmicutes*/*Bacteroidetes* ratio, and both actions could neutralize obesity progression and diminish body weight [47]. Finally, the duration of the exercise should change the intestinal microbiota composition, especially in beneficial-related bacteria, and as a consequence, permeability intestinal [58]. There is no detailed information regarding the ideal duration of the exercise, how the exercise could interact with the diet, and how other microbes (Archaea, viruses, and fungi) could be influenced by the duration of the exercise. Those issues remain unclear, and further studies are required.

### 4.3. Effects of Exercise Intensity on Microbiota

Several studies mention that aerobic exercise can be a beneficial strategy for modulating the microbiota composition in the presence of metabolic diseases, specifical exercises of moderate or vigorous intensity [59]. It has also been reported that stress induced by intense exercise increases intestinal inflammation and an increase in *Ruminococcus gnavus*, as well as *Butyrivibrio, Coprococcus*, and *Oscillospira,* and a decrease in *Turicibacter* spp. [17].

In athletes who practice intense and prolonged exercises, they report a particular microbiota composition, described by a bigger abundance of bacteria involved in inflammatory processes, such as *Haemophilus* and *Rothia* [60], *Mucispirillum* [61,62], and *Ruminococcus gnavus* [63]. *Faecalibacterium* abundance, generally recognized as favorable to human health [64,65,66], has been detected concurrently with an excessive pro-inflammatory abundance of bacteria in endurance runners whose abnormal gut environment may cause it to act as an opportunistic bacterium [18]. Finally, in a recent RCT [67], a reduction in microbial heterogeneity was observed in the intense-exercise group versus the control group.

On the contrary, it has been studied that intense exercise can reduce intestinal inflammation by changing the microbial profile [68]. An increase in *Bacteroidetes* has also been observed, which might be helpful to athletes by having a crucial role in the metabolic conversion of protein, complex sugar polymers degradation [4,68,69,70,71], improvements in glucose metabolism, and branched-chain amino acid degradation [72,73,74,75]. Another study defined that high-intensity exercise increases mitochondrial function and grows essential bacteria in urease production and lactate metabolism [76].

Likewise, a study showed that high-intensity interval training and resistance work modified the composition of the intestinal microbiota [77]. Regarding resistance work, it is capable of modestly modifying the microbiota composition and function compared to other types of exercise [78].

**Figure 2:**
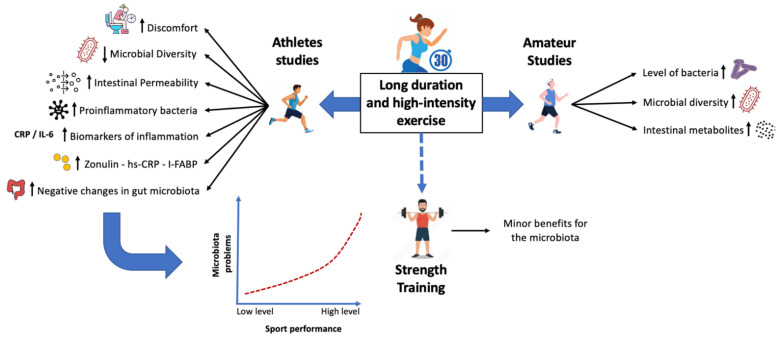
Interaction of different intensities and duration of exercise on the intestinal microbiota. Abbreviations: CRP, C-reactive protein; hs-CRP, high-sensitivity C-reactive protein; I-FABP, intestinal fatty-acid binding protein; IL, interleukin.

Regarding moderate-intensity exercise, a study in obese mice showed alterations in the gut microbiota of the colon and effective activation of the AMPK/CDX2 signaling pathway to improve the intestinal barrier [79]. In this sense, this has defined a correlation between cardiorespiratory fitness and greater microbial diversity in healthy subjects; therefore, enhanced cardiovascular fitness and oxygen consumption correlate positively with a more diverse microbial profile [80]. In accordance with the above, it has also been studied that subjects with low aerobic capacity display a greater *Eubacterium rectale-Clostridium coccoides* presence, related to metabolic disorders [81,82]. However, moderate-low intensity training has also been indicated to produce limited gut microbiota changes, and higher intensity appears to be essential in provoking changes in obese and overweight subjects [50]. Some specific microbial genera were related to specific diets and exercise-induced regulation of cardiometabolic health [83]. Figure 2 summarizes the main findings in the present systematic review.

### 4.4. Limitations of the Study

This study has found some limitations. The first of these is that not all the studies were controlled trials. Furthermore, only 7 of the 16 studies are of high quality. This makes interpretation difficult and increases bias. Therefore, caution should be exercised in interpreting the data obtained in this review. In addition, a combination of methods of measuring the quality of the articles had to be carried out based on the type of study. That is, one type of criterion was used for a cross-sectional study, another criterion for controlled trials, another criterion for quasi-experimental, etc. Finally, the studies used different methods to obtain a microbiota marker. Therefore, the state of the microbiota can be different depending on the method used.

## 5. Conclusions

Using the main findings of the present systematic review, it can be established that a large part of the studies found reported adverse effects on the intestinal microbiota when performing endurance exercises, such as an increase in distress, bacteria, a decrease in microbial diversity, and an intestinal permeability decrease. However, the rest of the studies found positive effects with aerobic exercise. In addition, in the studies carried out on athletes, more negative effects on the microbiota were found than in those carried out on non-athletic subjects. It was observed that strength training obtains the lowest benefits to the microbiota. In general, it is appreciated that the studies obtain molecules that favor the microbiota and other pro-inflammatory elements at the same time. This leads us to think that there is no absolute clarity of the mechanisms and personal and environmental factors that influence an improvement or worsening of the microbiota as a function of exercise. Future studies should propose what is the amount of exercise that must be achieved in order to have favorable effects on the microbiota and what is the cut-off point in the dose of exercise that begins to worsen the conditions of the intestinal microbiome.

## Figures and Tables

**Table 1:** Search strategy in databases.

Database	Search Strategy	Limits	Filters
Web of Science	(ALL (Physical activity AND gut microbiota OR Physical activity AND intestinal barrier OR Physical activity AND intestinal permeability OR Physical exercise AND gut microbiota OR Physical exercise AND intestinal barrier OR Physical exercise AND intestinal permeability))	Title Articles English	238 items filtered
PubMed	(Physical activity OR physical exercise) AND (gut microbiota OR intestinal barrier OR intestinal permeability)	Title Articles English Humans	104 items filtered
Scopus	TITLE-ABS-KEY (physical AND activity AND gut AND microbiota) OR (physical AND activity AND intestinal AND barrier) OR (physical AND activity AND intestinal AND permeability) OR (physical AND exercise AND gut AND microbiota) OR (physical AND exercise AND intestinal AND barrier) OR (physical AND exercise AND intestinal AND permeability) AND (LIMIT-TO (OA, “all”)) AND (LIMIT-TO (PUBYEAR, 2022) OR LIMIT-TO (PUBYEAR, 2021) OR LIMIT-TO (PUBYEAR, 2020) OR LIMIT-TO (PUBYEAR, 2019) OR LIMIT-TO (PUBYEAR, 2018) OR LIMIT-TO (PUBYEAR, 2017) OR LIMIT-TO (PUBYEAR, 2016) OR LIMIT-TO (PUBYEAR, 2015)) AND (LIMIT-TO (DOCTYPE, “ar”)) AND (LIMIT-TO (LANGUAGE, “English”))	Title Articles English	5934 items filtered

**Table 2:** Characteristics as the type of study, aim, sample, design, and mean results of the studies.

Author, Year	Type of Study	AIM	Sample	Study Design	Results
Pugh et al. (2017) [5]	Quasi-Experimental	Characterize the HIIT effects on small intestinal damage markers	*n* = 11 (men runners trained) Aged 33.1 ± 10.4; VO_2max_ 60.0 ± 3.2 mL/kg/min	Acute HIIT episode markers of intestinal permeability and damage were evaluated and compared with resting conditions. Minimum running performance of 10 km (39 min) and a minimum of 5 workout sessions per week, using serum sampling, pre-exercise, after each set of exercises, and 2 h post-baseline	HIIT significantly increased the serum lactulose: rhamnose ratio and sucrose concentrations compared with rest. In contrast, urinary lactulose: rhamnose or sucrose concentrations did not vary between study groups. Plasma I-FABP augmented in the recuperation period from HIIT only. After 24 h of HIIT, the researchers found mild symptoms of GI distress
Liang et al. (2019) [9]	Cross-sectional	Whether the intestinal microbiota is distinctive between higher-level and lower-level athletes	*n* = 31 (professional martial arts athletes). 15 women and 16 men; aged 20–24	Martial arts athletes; Wushu routine, vigorous, fast and dynamic sports. The researchers used 16S rRNA gene sequencing to determine the intestinal changes	Higher-level athletes have augmented metabolic capacity and diversity in the intestinal microbiota compared with lower-level athletes.
Petersen et al. (2017) [7]	Cross-sectional	Determine the presence of distinctive organisms in professional and amateur level competitive cyclists	*n =* 33 (professional and amateur level competitive cyclists); 11 women and 22 men; aged 19–49	The study used metatranscriptomic (RNA-Seq) sequencing and mWGS	The increase in *Prevotella* was associated with time reported exercising during an average week. Several professional cyclists have augmented levels of *Methanobrevibacter smithii* transcripts compared with amateur cyclists.
Bressa et al. (2017) [27]	Cross-sectional	Compare intestinal composition among two groups divided by physical exercise levels	*n* = 40 (premenopausal women). 19 active and 21 sedentary Aged 18–40; BMI 20–25 kg/m^2^	The researchers used 16S rRNA gene sequencing to determine the intestinal changes	Performance of physical activity was associated with the presence of health-promoting bacteria (*R. hominis*, *A. muciniphila, Bifidobacterium* spp., and *F. prausnitzii*). Decreased levels of diversity were correlated with sedentary parameters
Karhu et al. (2017) [16]	Quasi-experimental	Evaluate the effect of running on GI function markers	*n* = 17 (active runners); 8 women and 9 men; aged 18–45	The researchers measured secondary variables, such as zonulin, levels of serum intestinal I-FABP, and bacterial LPS, among others	Both, serum I-FABP and intestinal permeability increased after running, without differences amongst groups. No changes were observed in the bacterial LPS in serum
Keohane et al. (2019) [13]	Long-term	Analyze the changes in the intestinal microbiota of four well-trained male athletes to prolonged, high-intensity trans-oceanic rowing	*n* = 4 (men athletes transatlantic rowing). Aged 25–27; BMI 23–25 kg/m^2^; VO_2Max_ 46–50 mL/kg/min	Metagenomic whole-genome shotgun sequencing was used	Intense exercise clearly impacts the diversity of the intestinal microbiota, with changes in specific bacteria related to metabolic pathways
Bycura et al. (2021) [31]	Quasi-experimental	Impact of CRE or RTE on intestinal microbiota	*n* = 56 *n* = 28 CRE group (21 women; Aged 20.7; BMI 24.5 kg/m^2^ and 7 men; aged 20; BMI 24.0 kg/m^2^. *n* = 28 RTE group (17 women; aged 20.4; BMI 23.2 kg/m^2^ and 11 men; aged 22.6; BMI 24.59 kg/m^2^	Intestinal microbiota was measured using 16S rRNA gene sequencing	The observed changes were associated only with the CRE group, resulting in disturbance of the intestinal microbiota
Morishima et al. (2020) [18]	Cross-sectional	Effects of highly intensive endurance exercise on the intestinal microbiota and its relationship with the onset of the exercise-induced GI disorders	*n* = 29 (15 women Japanese endurance runners and 14 nonathletic but healthy women). Aged 20–21; BMI 20.7–21.9 kg/m^2^	Fecal microbiota was tested using 16S rRNA metagenomics, and other variables such as moisture content, organic acids, and putrefactive metabolites concentrations were examined	Female elite endurance runners have more abundance of *Faecalibacterium*, and these changes could be associated with the succinate concentration in this group
Tota et al. (2019) [19]	Long-term	Evaluate intestinal and muscle damage in triathletes	*n* = 15 (triathletes). Aged 6–14; VO_2max_ 58.8 ± 4.5 mL/kg/min	Variables used for the analysis were: cortisol, c-reactive protein, zonulin, and TNF-α	Zonulin and variables of permeability were augmented after the race
Zhao et al. (2018) [22]	Quasi-experimental	The gut microbiota immediately responds to the enteric changes in amateur half-marathon runners	*n* = 20 (4 women and 16 men amateur half-marathon runners). Aged 31.3; BMI 22.6 kg/m^2^	Fecal samples were analyzed before and after the marathon using 16 rDNA sequencing analyses	*Coriobacteriaceae* changes were related to the exercise role in avoiding disease and refining health outcomes.
Moitinho-Silva et al. (2021) [34]	Randomized controlled trial	Analyze the changes in the intestinal microbiota on previously physically inactive, healthy adults in comparison to controls that did not perform regular exercise	*n* = 36 (11 controls; 13 endurance group; 12 strength group). Aged 22–41.3; BMI 19.7–32.5 kg/m^2^	Fecal microbiota was tested using 16S rRNA metagenomics	Mucosal damage and inflammation were found after short-term resistance training. No changes were observed in intestinal microbiota
Sadowska-Krepa et al. (2021) [29]	Quasi-experimental	Evaluate intestinal damage in middle-aged male subjects	*n* = 10 (amateur long-distance runners). Aged 21–35	Variables used for the analysis were: TAS, TOS/TOC, hs-CRP, I-FABP, and zonulin	After the exercise, the levels of intestinal permeability biomarkers as, hs-CRP, I-FABP, zonulin, and inflammation were augmented
Kulecka et al. (2020) [33]	Quasi-experimental	Evaluate differences in intestinal microbiota amongst healthy controls and endurance athletes	*n* = 71 *n* = 14 marathon runners; *n* = 11 cross-country skiers; *n* = 46 healthy control individuals	Fecal microbiota was tested using 16S rRNA metagenomics	Excessive training is associated with changes in *Bacteroides* and *Prevotella* and bacterial diversity
Tabone et al. (2021) [30]	Quasi-experimental	Determine whether the changes are driven by exercise on the gut microbiota (with 16S rRNA gene) and the serum and fecal metabolome	*n* = 40 (men endurance cross-country runners). Aged 35.8 ± 8.0; BMI 22.8 ± 2.1 kg/m^2^; VO_2max_ 58.8 ± 3.24 mL/kg/min	Fecal microbiota was tested using 16S rRNA metagenomics	The changes in gut microbiota could be related to physiological changes in ammonia, uric acid, and lactate
Barton et al. (2017) [28]	Cross-Sectional	Evaluate differences in intestinal microbiota amongst exercise and a more sedentary state	*n* = 86 (40 men professional international rugby union players and 46 men controls)	Fecal microbiota was tested using 16S rRNA metagenomics	Professional international rugby union players had more favorable effects in metabolic pathways than the control group
Craven et al. (2021) [32]	Quasi-experimental	Evaluate differences in intestinal microbiota according to training volume	*n* = 14 (highly trained middle-distance runners). *n* = 6 women; aged 22.0 ± 3.4; VO_2max_ 59.0 ± 3.2 mL/kg/min *n* = 8 men; aged 20.7 ± 3.2; VO_2max_ 70.1 ± 4.3 mL/kg/min	Fecal microbiota was tested using 16S rRNA metagenomics	No changes were observed in intestinal microbiota according to training volume in upper taxons. Changes in family, genus, and species were observed, these changes did not return to pre-levels

Abbreviations: AFT, after fecal; BEF, before fecal; BMI, body mass index; CRE, cardiorespiratory exercise; DS, standard deviation; FCCS, female cross-country skiers; FDR, false discovery rate; FMR, female marathon runners; GI, gastrointestinal; HIIT, high-intensity interval training; hs-CRP, High-sensitivity C-reactive protein; HvolTr, high-volume training; I-FABP, intestinal fatty acid-binding protein; kg/m^2^, kilogram per square meter; LPS, lipopolysaccharide; MCCS, male cross-country skiers; MCHC, mean corpuscular hemoglobin concentration; mL/kg/min, milliliters per minute per kilogram; MMR, male marathon runners; mWGS, metagenomic whole genome shotgun; NormTr normal training; PGM, personal genome machine; PWC, physical working capacity; rRNA, ribosomal ribonucleic acid; RTE, resistance training exercise; TaperTr, exponential reduction in training; TAS, total antioxidant status; TOC, total oxidant capacity; TOS, total oxidant status; VO_2max_, the maximum amount of oxygen; WHO, World Health Organization; WSER, Western States Endurance Run.

**Table 3:** Checklist from Joanna Briggs Institute’s criterium according to kind of study, percentage of criterium reached, and quality level of evidence.

Criteriums According to Kind of Study															
Authors	1	2	3	4	5	6	7	8	9	10	11	12	13	Percentage Reached	Quality Level
Pugh et al. [5]	1	0	0	0	1	1	1	1	1					66.7	MQ
Liang et al. [9]	0	1	1	1	1	0	1	1						75.0	HQ
Petersen et al. [7]	0	1	1	1	1	0	1	1						75.0	HQ
Bressa et al. [27]	1	1	1	1	1	1	1	1						100.0	HQ
Karhu et al. [16]	1	0	0	0	1	1	1	1	0					55.6	MQ
Keohane et al. [13]	0	1	1	1	1	1	1	1						87.5	HQ
Bycura et al. [31]	1	1	1	0	1	1	1	1	1					88.9	HQ
Morishima et al. [18]	0	0	1	1	1	0	0	1						50.0	MQ
Tota et al. [19]	0	1	1	1	0	0	1	0						50.0	MQ
Zhao et al. [22]	0	0	0	0	1	1	1	1	1					55.6	MQ
Moitinho-Silva et al. [34]	1	0	1	0	0	0	1	1	1	1	1	1	1	69.2	MQ
Sadowska-Krepa et al. [29]	1	0	0	0	1	1	0	1	1					55.6	MQ
Kulecka et al. [33]	0	1	1	1	1	1	1	1	1					88.9	HQ
Tabone et al. [30]	1	0	0	0	1	1	1	1	1					66.7	MQ
Barton et al. [28]	1	0	1	1	0	1	1	1						75.0	HQ
Craven et al. [32]	0	0	0	0	1	1	1	1	1					55.6	MQ

HQ: high quality; MQ: medium quality.

## Data Availability

Not applicable.
